# MALDI-Imaging Mass Spectrometry: a step forward in the anatomopathological characterization of stenotic aortic valve tissue

**DOI:** 10.1038/srep27106

**Published:** 2016-06-03

**Authors:** Laura Mourino-Alvarez, Ibon Iloro, Fernando de la Cuesta, Mikel Azkargorta, Tamara Sastre-Oliva, Iraide Escobes, Luis F. Lopez-Almodovar, Pedro L. Sanchez, Harkaitz Urreta, Francisco Fernandez-Aviles, Angel Pinto, Luis R. Padial, Finn Akerström, Felix Elortza, Maria G. Barderas

**Affiliations:** 1 Department of Vascular Physiopathology, Hospital Nacional de Parapléjicos, SESCAM, Toledo, Spain; 2 Proteomics Platform, CIC bioGUNE, CIBERehd, ProteoRed-ISCIII, Bizkaia Science and Technology Park, Derio, Spain; 3 Cardiac Surgery, Hospital Virgen de la Salud, SESCAM, Toledo, Spain; 4 Department of Cardiology, Hospital Universitario de Salamanca-IBSAL, Salamanca, Spain; 5 Department of Cardiology, Hospital General Universitario Gregorio Marañón, Madrid, Spain; 6 IDEKO-IK4, Arriaga Industrialdea, Elgoibar, Spain; 7 Cardiac Surgery, Hospital General Universitario Gregorio Marañón, Madrid, Spain; 8 Department of Cardiology, Hospital Virgen de la Salud, SESCAM, Toledo, Spain

## Abstract

Aortic stenosis (AS) is the most common form of valve disease. Once symptoms develop, there is an inexorable deterioration with a poor prognosis; currently there are no therapies capable of modifying disease progression, and aortic valve replacement is the only available treatment. Our goal is to study the progression of calcification by matrix-assisted laser desorption ionization imaging mass spectrometry (MALDI-IMS) and get new insights at molecular level that could help in the understanding of this disease. In this work, we analyzed consecutive slices from aortic valve tissue by MALDI-IMS, to establish the spatial distribution of proteins and peptides directly from the surface of the histological sections. The analysis showed different structures corresponding to regions observed in conventional histology, including large calcification areas and zones rich in collagen and elastic fibers. Peptide extraction from the tissue, followed by liquid chromatography mass spectrometry analysis, provided the identification of collagen VI α-3 and NDRG2 proteins which correlated with the masses obtained by MALDI-IMS and were confirmed by immunohistochemistry. These results highlighted the molecular mechanism implied in AS using MALDI-IMS, a novel technique never used before in this pathology. In addition, we can define specific regions proving a complementary resolution of the molecular histology.

Aortic valve stenosis (AS) is now the most common valvular heart disease and the third most common cardiovascular disease affecting mainly people who are over 65 years of age. Aortic stenosis is the result of aortic valve (AV) narrowing resulting in a reduced AV area, producing blood flow obstruction with a pressure gradient between the left ventricle (LV) and ascending aorta^1^. With increased LV pressure there is increased myocardial wall stress, which is initially compensated by LV hypertrophy, followed by LV dilatation and systolic dysfunction in the latter stages of the disease. During the initial phase, the disease remains silent, however, when AS becomes severe a classical triad of symptoms follows (angina, syncope and dyspnea), at which point AV replacement is indicated to alleviate symptoms and reduce mortality^2^,^3^. In Occident, the most common etiology of AS is calcific aortic stenosis (CAS), which progression involves destruction of the endothelial layer overlying the valve, followed by infiltration of inflammatory cells, such as monocytes, T lymphocytes and mast cells. In addition, these valves accumulate oxidized lipids and apolipoproteins and, as the disease progresses, matrix remodeling and active bone formation is observed^4^. Noteworthy, the speed at which AS progresses varies significantly from patient to patient for which reason clinical practice guidelines recommend regular follow-up with echocardiography even at the earlier stages of the disease^5^,^6^.

A tissue proteomic study was carried out previously in our lab to shed light onto AS molecular mechanisms^7^. Nevertheless, this study did not allow the localization of the proteins/peptides in a specific area of the tissue neither the follow-up of the lesion along the valve. To solve this limitation, we present now a more focused study in which we have followed the progression of the lesion on tissue with the purpose of elucidating specific molecular mechanisms involved in CAS and novel proteomic alterations. We believe that increased knowledge of the CAS disease process should allow us to: a) develop drugs that target a specific pathophysiological molecular mechanisms in CAS and; b) discover biomarkers which may guide the clinician to classify patient into high/low risk of disease progression and subsequently tailor follow-up frequency, thus optimizing healthcare resources and reducing costs.

To date, there are several technologies available to analyze the proteins present in a tissue, such as two dimensional gel electrophoresis mass spectrometry (2DE-MS), or direct liquid chromatography (LC)-MS, but in both cases, the main drawback is that sample preparation removes the direct relationship between morphological tissue regions and a specific protein localization. The use of MALDI-imaging mass spectrometry (MALDI-IMS), a novel proteomic tool, offers the great advantage to investigate the physiopathological changes taking place directly in tissue while retaining the histopathological context, allowing the simultaneous mapping of hundreds of peptides and proteins present in tissue sections with a lateral resolution of approximately 50–75 microns. MALDI-IMS images show the spatial spread of a particular peak’s height over the tissue, which can be extrapolated to the amount of each particular ion that was measured. The key point is that analyte signals can be correlated with underlying tissue architecture without any geometrical distortion, enabling the so-called “Molecular Histology”^8^,^9^. This technique has enormous applications in clinical medicine. For example, pathologists are able to correlate the distribution of specific compounds with pathologically interesting features^10^,^11^,^12^. Recent work has also demonstrated the capacity to create three-dimensional molecular images using the MALDI-IMS technology and co-registration of these image volumes to other imaging modalities such as magnetic resonance^13^,^14^.

In this work, we present that novel MALDI-IMS analysis applied to 3 human AV tissue sections of the same valve, with different injury severity, revealed the existence of different regions which correlate to conventional histology. In addition, we have seen that some peptides were shown to define specific regions not evident in conventional histology, suggesting a higher resolution and the complementarities of both techniques. Peptides coming from collagen VI α-3 (CO6A3) and N-myc downstream-regulated gene 2 protein (NDRG2) proteins were identified and further confirmed by immunohistochemistry (IHC). Noteworthy these, two proteins have been previously related with matrix mineralization and apoptosis, which are mechanisms involved in the development of the CAS.

## Results

### MALDI-IMS analysis

**Figure 1:**
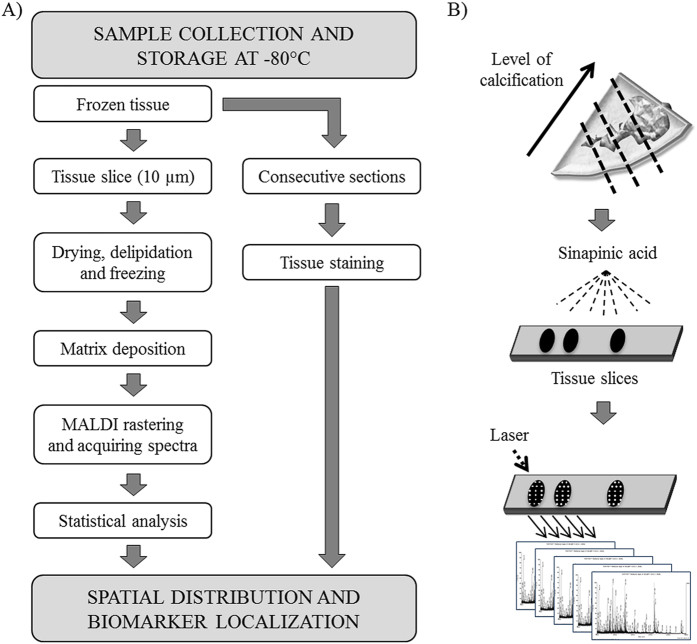
(**A**) Schematic representation of MALDI-IMS protocol. Samples are collected and immediately frozen at −80 °C. Subsequently, frozen tissue is included in water and cut using a cryostat. Sections are then dried and delipidized prior to MALDI-IMS analysis. Besides, consecutive sections are also collected for histological staining. (**B**) Schematic representation of tissue sections and subsequent MALDI-IMS analysis. Three different sections with different degree of calcification along the tissue were taken for analysis and sprayed with sinapinic acid matrix. Afterwards, sections were analyzed using MALDI-IMS. As shown in the scheme, the laser runs through the whole sections surface and one spectrum is generated for each laser shot.

MALDI-IMS was performed on tissue sections of AV at three different levels of the calcified lesion. The performed workflow is summarized in Fig. 1. The analysis of each data set included molecular mass images, principal component analysis (PCA) and hierarchical clustering. Following the MALDI-IMS experiment, consecutive sections were stained with H&E, Van Giesson, oil red or immunohistochemically (Figure SM1). This allowed a parallel assessment of the histopathological features observed under the light microscope and the proteome profiles obtained by MALDI-IMS.

**Figure 2:**
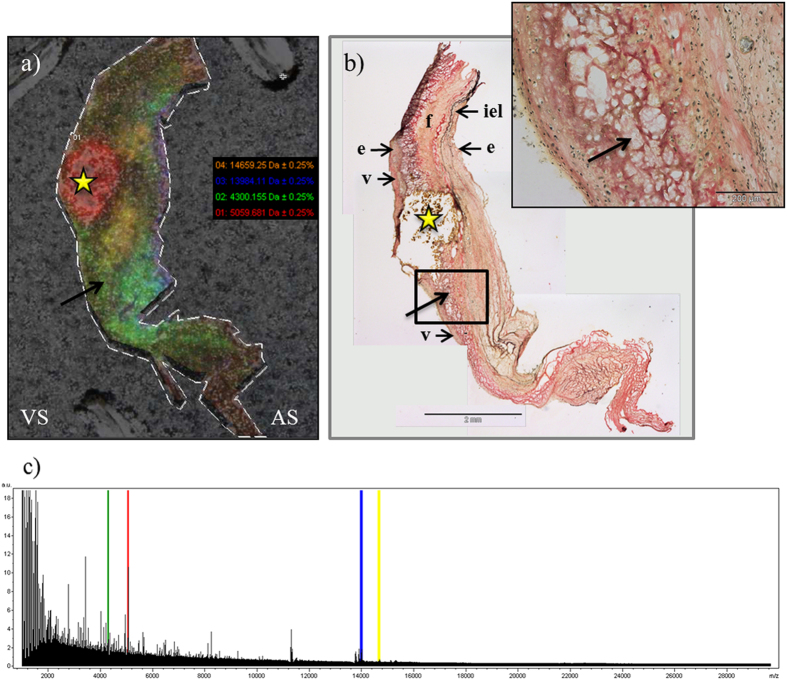
(**a**) Spatial distribution of the selected peaks. (**b**) Verhoeff-Van Giesson staining for elastic fibers. In this staining we can observe the same than in (**a**), which correspond with a MALDI-IMS image: the existence of a prominent and evident calcification area which displaced the elastic lamina. (**c**) Corresponding average spectrum of the previous MALDI-IMS image. Good signals in both achieved resolution and intensity are clearly seen. In this panel we can compare mass spectrometry imaging with immunohistochemistry image. As a result, we can highlight the correspondence in the calcified zone, rich in fragments of 5059 Da (red area, star). Besides, it also exists a correlation between collagen-rich areas and the 4300 Da peak (enlarged image, green area, arrow), and elastic-fibers-rich area with the 13984 peak. VS: ventricular side; AS: aortic side; e: endothelium; iel: internal elastic lamina; f: fibrosa layer; v: ventricular layer.

For a practical experiment, a balance must be made between reasonable data acquisition size, spatial resolution, and the time spent on acquiring MS imaging data. For AV tissue we performed several trials with different lateral resolution (data not shown): 100 µm and 75 µm in order to find the best lateral resolution/signal intensity ratio (with our equipment, 75 µm represent the best ratio). Once the set of mass spectra from a MALDI-IMS experiment has been acquired, the image for each of the detected ions is generated, with each pixel representing a laser irradiation spot from the surface of the tissue section. The combination of all the individual pixels with different ion intensities across the tissue surface reflects the ionization of target analytes within the tissue. From a technical point of view, obtained MALDI-IMS results showed a good lateral resolution (75 μm) and extraction ratio, with practically no evidence of significant lateral diffusion, and showing up to 140 analyte signals across the mass window up to 20000 Da, with satisfactory intensities (signal to noise ratio >3) (Fig. 2c).

MALDI-IMS images evidenced different structures that correspond to areas seen in conventional histology. In Fig. 2, where the less affected tissue section is shown, 4 ion signals were selected manually according to their distribution in characteristic regions of the lesion, including large calcification areas (red, 5059 Da), collagen-rich areas (green, 4300 Da) and elastic fibers-rich areas (blue, 13984 Da). The two prominent zones of 14659 Da peaks (yellow) are not evident with conventional staining, showing the capabilities of this technique in discovering subjacent molecular patterns when compared with other techniques.

**Figure 3:**
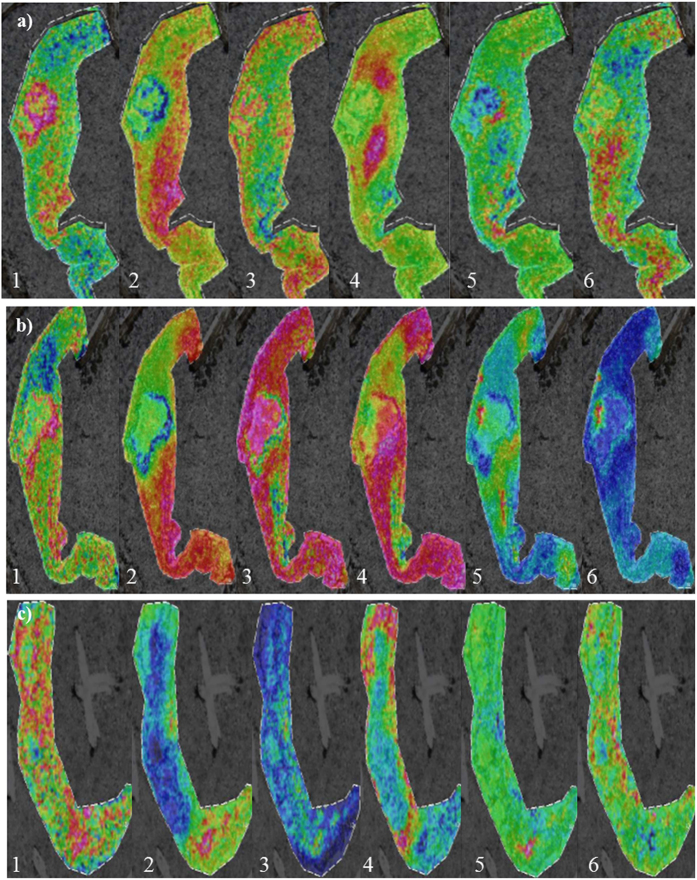
PCA images: In this panel we show the 6 most informative PC (1 to 6) of each analyzed section. Different colors show different regions according to the distribution of the peaks. (**a**) corresponds to the PCA analysis of the first section, (**b**) correspond to PCA of the second section and (**c**) correspond to the PCA analysis of the third section, always ordered in increasing level of affectation of the AV.

**Figure 4:**
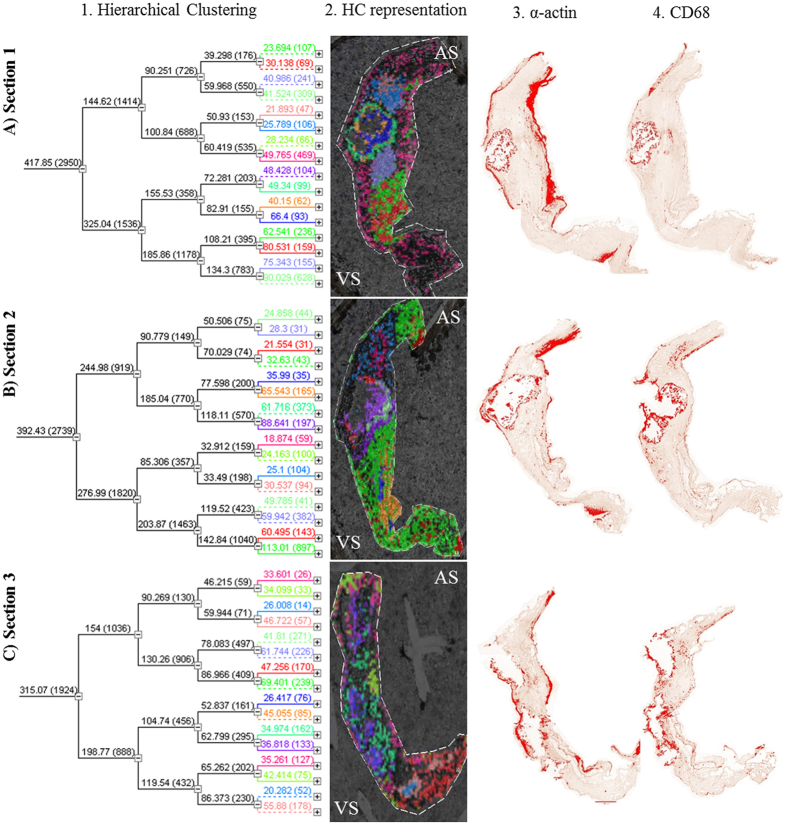
Results from hierarchical clustering and IHC of the three analyzed sections. (**A**) less affected tissue; (**B**) intermediate affected tissue; (**C**) severe affected tissue). (**1**) The dendrogram resulting from the hierarchical clustering shows the distances between the resulting clusters (degree of similarity or dissimilarity between clusters [spectra groups]. The spectra from the different calcification areas are similar, as we would expect, although is possible to distinguish the different regions. (**2**) Different colours in the hierarchical clustering images show different histological features at a molecular level. (**3**), (**4**) Red areas in the IHC images correspond to positive staining according to ImageJ analysis for myofibroblasts (α-actin) (3) and macrophages (CD68) (4) staining. VS: ventricular side; AS: aortic side.

In an effort of making the analysis as unbiased as possible, when we studied the underlying histological features, an unsupervised feature extraction by PCA of MALDI-IMS data was performed. Briefly, PCA is a well-known method used to describe the variability in the data set in a reduce number of dimensions. At the end, PCA can remove those components that are not significant according to the underlying structure, while reconstructs images that contains most of the information^15^,^16^, facilitating the exploration of the usually complex structures that exist in tissues. Up to 8 PC of each section were extracted (PC1 to PC8) according to the variance of the mass spectra in the surface of the tissue (Fig. 3). Calcified nodule shows the most different peptide pattern, so is perfectly separated in the three sections (PC1 and 2). Besides, there are different peptide patterns, not only at both sides of the AV (corresponding with the different histological layers) but also revealing different structures surrounding calcification areas. Right after, hierarchical clustering, which allows the unsupervised classification of the mass peaks in the imaging data set by spectra similarity, was performed. Using this algorithm it is possible to explore the hierarchical relationships between analyte signal classes that correspond to histological features present on analyzed tissue in the sample (Fig. 4). For this analysis, we have used the PCA data, so the clustering will be more focused on the obvious differences in the data set, avoiding fluctuations that may happen in a random way. In the first section, the areas coinciding with the ventricular and the subendothelial layer have a similar molecular pattern (pink region), which is essentially different from the central area of the valve. Furthermore, the calcified area is perfectly separated from the rest of the tissue and two adjacent regions to the calcification (dark blue and dark green). It may be related to the progression of the lesion, as in consecutive sections they appear as calcified nodules. It is noticeable the concentric layers that appears around the calcification. These regions belong to the same branch of the hierarchical tree (Figure 4.1.a), which means that they are related but significant differences at molecular level exist between them. In the second section, the differentiation of the calcified zone (purple region) is maintained while the layered structure observed in the first one has disappeared. In the third section, dispersed structures at a molecular level are observed.

### Identification of MALDI-IMS obtained masses by nLC-MS/MS

With the aim of correlating the masses obtained by MALDI-IMS to the corresponding protein identifications, we have performed a peptide isolation approach followed by nLC-MS/MS. In this approach, a mild peptide/protein extraction was performed using the same conditions as in MALDI-IMS in order to extract only those analytes that could be observed by MALDI.

The ions in the range of 800–4500 Da. which happened to be informative in the stenotic progression were confronted against the identifications obtained in nLC MS/MS. As a result, we identified two peptides corresponding to CO6A3 and NDRG 2 (Table 1).

### Immunohistochemistry

**Figure 5:**
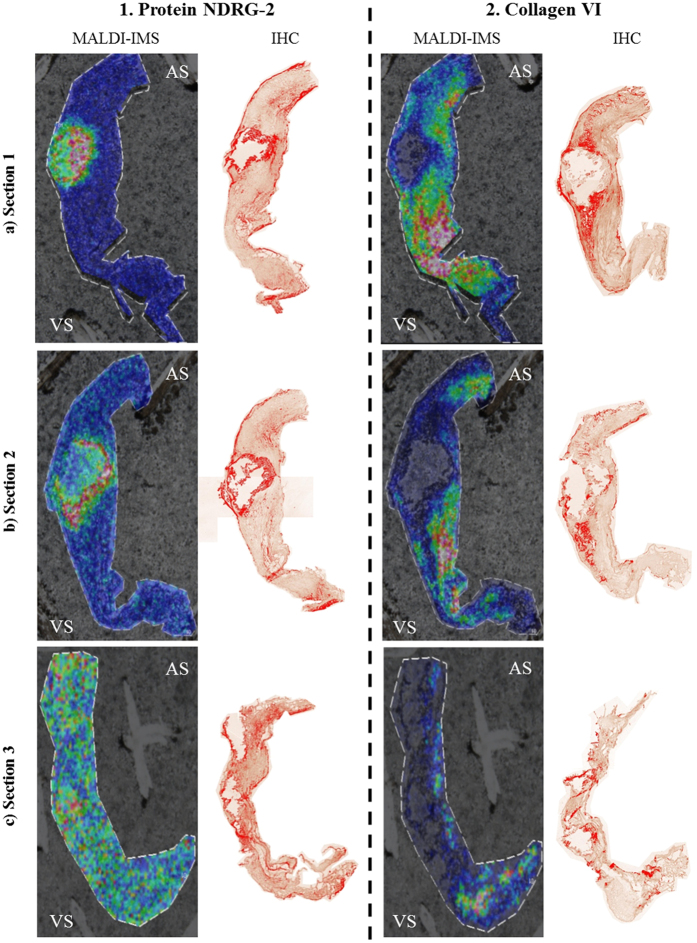
Comparison between MALDI-IMS images and IHC. (1) NDRG-2 and (2) and CO6A3 of the three analyzed sections: (**a**) less affected tissue; (**b**) intermediate affected tissue; (**c**) severely affected tissue. In the MALDI-IMS images it is shown the superficial distribution from identified peptides: AELQEVQITEEKPLLPGQTPEAAKEAELAA for NDRG-2 and IEEGVPQFLVLISSGKSDDEVDDPAVELKQFGVAPFTIAR for CO6A3. Different colors indicate the abundance of the peptide from pink (the most abundant) to dark blue (the less). In the IHC images, the positive staining for these proteins according to the ImageJ analysis it is shown in red. It is visible the correlation between the NDRG-2 staining and the corresponding peptide. On the contrary, in the case of collagen IV, the more intensity of the peak, the less protein signal in the IHC, maybe due to the degradation that is suffering this protein. VS: ventricular side; AS: aortic side.

MALDI-IMS allows the spatial localization of peptides. Since antibodies recognizing the epitope in this defined sequence were not available, we proceeded to the detection of the proteins that the peptides belonged to. Therefore, adjacent sections from the one analyzed in molecular histology were used to confirm the proper identification and co-localization of each protein with the corresponding detected peptide (Fig. 5).

According to the molecular histology and the ImageJ analysis, we can observe that the distribution of the 3398 Da peak overlap with IHC signal of protein NDRG-2. This protein is condensed in the margins of the calcification area but also expressed at lower levels along all the surface of the sections, showing a good co-localization with the peptide. On the contrary, in the case of CO6A3, the more intensity of 4321 Da peak, the less protein signal in the IHC was observed. Considering that we analyzed peptides in the molecular histology and complete proteins in the IHC, this could be due to degradation of CO6A3, as we will discuss later. It is important to point out that these correlations are easily observed in the first and second sections of the IHC; nevertheless, in the third one, is difficult to find a good correlation due to the structural damage that suffers the tissue when the calcification advances.

**Figure 6:**
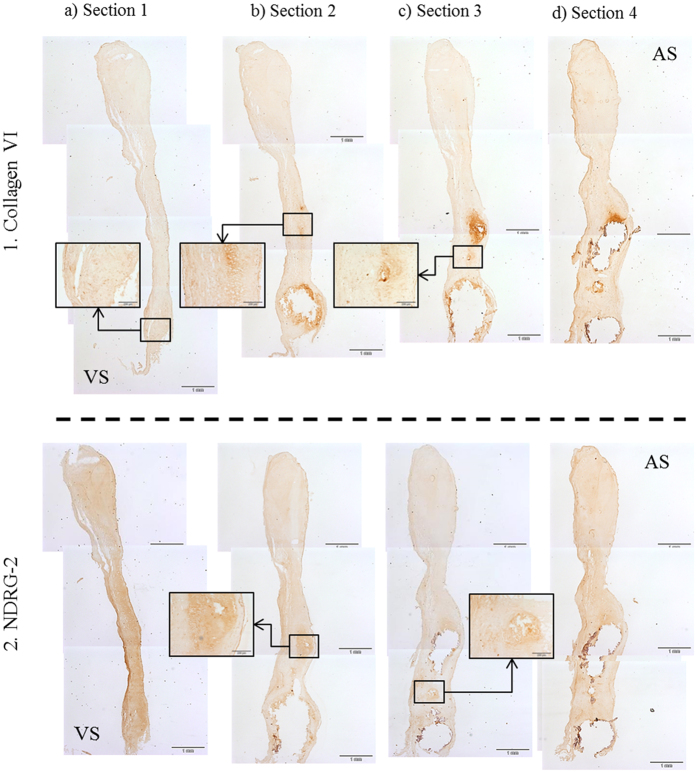
Results from (1) CO6A3 and (2) NDRG-2 staining using several sections with different degrees of calcification (from mild to severe lesion: a–d) of a stenotic valve. Positive staining (darker brown) is observed surrounding the calcification areas (enlarged images) which is consistent with an implication of these proteins in the calcification process. VS: ventricular side; AS: aortic side.

Additionally, several sections of 4 different AV were used to follow the superficial distribution of CO6A3 and NDRG2 along the different stages of the lesion (Fig. 6). We can observe that, for both proteins, positive staining appears mainly around the calcified area.

## Discussion

MALDI-IMS analysis allowed us to study the surface distribution of endogenous peptides of the stenotic AV tissue^17^,^18^. This methodology offers several advantages over other imaging methods like autoradiography, such as the high specificity of MS detection, minimal sample preparation, applicability to a wide variety of analytes and multiplexing capability. These advantages can be summarized in the possibility to obtain molecular maps while preserving their original location in the tissue. As drawbacks, it is only capable of detecting rather abundant proteins and peptides, because of the absence of a prefractionation procedure. Besides, the images correspond to the addition of thousands of different spectra, so a considerable effort is required for the analysis although nowadays entire MS acquisition is performed in automatic fashion. It should be also noticed the technical limitation related to mass range acquisition that affects the identification process due to the limited m/z window. In this work, after the updating of the technique with these kinds of samples, three different sections of the same valve with growing severity were studied with the objective of locating differences at a molecular level in the different states of the lesion. In order to classify the AV sections, histological staining for elastic fibers and lipids were performed, as well as IHC for detection of macrophages and myofibroblasts.

As the lesion in CAS progresses, tissue degradation increases due to the extension of the calcified region, which affects the integrity of the tissue^19^. Due to this feature of CAS, the resolution of molecular images and the quality of histologies of the first and second section were much better than in the third, which is pathologically affected in higher degree. Furthermore, the layer structure of the AV present in the first section is gradually lost and is not observable in the third section, neither in the conventional nor the molecular histology.

We have performed an unsupervised feature extraction by PCA and hierarchical clustering analysis, which allows the unbiased analysis of different regions of the valve, and also a manual inspection of the MALDI-IMS peaks according to the observed regions using histological techniques. PCA is a way to project high dimensional data to lower dimensions while retaining essential information by reducing the number of original variables. This technique transforms the original variables defined by peaks intensities to new variables, which are now called principal components (PC) that better explains the variance in the dataset. This variable transformation is defined in such a way that the first new variable has the largest possible variance with the final purpose of removing those components that are not significant while reconstructing images that contains most of the original information. In this context, the PCA is an unsupervised feature extraction and allows the unbiased generation of meaningful tissue images without a detailed understanding of the underlying histology. This allows a blind analysis of the peaks in the tissue surface without any bias due to the previous knowledge about tissue structures and facilitates the exploration of these usually complex samples. This pattern distribution analysis shows several different zones such as the calcified nodule and the different structures surrounding it. Additionally, hierarchical clustering was performed to do an unsupervised classification of the mass spectra in an imaging data set by spectra similarity. It allows exploring the hierarchical relationships between the peaks in the surface of the tissue that correspond to histological features and it has a great potential for defining new unknown regions that have never been seen using directed techniques such as IHC. As expected, this analysis goes a step further than PCA by identifying several concentric layers surrounding the calcification area, differing in their peptide pattern (Fig. 4). These concentric regions are interestingly grouped in the same branch of the hierarchical tree (Figure 4.1.a) showing small but significant differences at molecular level. This result shows the potential of MALDI-IMS as it is able to detect new areas of interest and structural patterns. Further analyses of these regions could be revealing as identification of the peaks of these concentric regions may show how the calcification is formed and developed. Some of these regions identified by unbiased analysis (PCA and hierarchical clustering), and all of those manually analyzed revealed a clear correspondence between the images from histological staining and the molecular ones, especially in the first tissue section. Maybe the most noticeable is the identification of the calcified nodule in the multivariate analysis but it is also remarkable the differentiation of the central area of the valve from the outer layers, which present similar molecular patterns. Nevertheless, it is not possible to observe a direct correlation between the molecular histology images and the characteristic presence of myofibroblasts and macrophages (α-actin and CD68 staining, respectively) that occurs in CAS. On the other hand, it is also observable the loss of the tissue structure when the calcification is more severe. These results are consistent with the degenerative process of the CAS, as the tissue undergoes a severe disintegration as the lesion progresses. Besides, the definition of different areas around the calcification nodule and the delineation of a specific region shown by one the peaks selected manually (14659.25, yellow color in Fig. 2) supports the potential utility of MALDI-IMS to deepen in the histological characterization of AV tissue. Accordingly, this study has highlighted a higher resolution of MALDI-IMS which might be an alternative to traditional anatomopathological analyses using routine histological techniques. In the future, a comprehensive analysis of the areas of interest defined using MALDI-IMS may allow us to deepen in the mechanisms that take place during CAS which is the first step for developing new strategies for preventing and treating this disease.

Once the most informative analyte peaks were defined, we proceeded to their putative identification by nLC-MS/MS analysis. Peptides were extracted using the same solvents in which the matrix is prepared. The extracted peptides were subjected to nLC-MS/MS. It is remarkable that the observed peptides are naturally occurring peptides, which are more difficult to analyze due to different reasons: non tryptic, can be large, could suffer different modifications, etc.^20^. Moreover, some of them may result to be too large for regular nLC-MS/MS experiments and dedicated methods for the “middle-down” approach should be used. Of note is the fact that the mass observed by MALDI-IMS comes from the operation of the MALDI in linear mode, which also complicates the analysis. The irregularities of the tissue and the limitation of this measuring method render low mass accuracy for the observed analytes, whereas when performing the measurement in high resolution nLC-MS/MS, the mass accuracy is high (around 0.1% of the mass). Peptides corresponding to CO6A3 and NDRG-2, which are involved in the development of CAS^21^,^22^ were identified by this approach. Being aware of our limitations in the identification process, we performed an IHC analysis, with the corresponding commercial antibodies, in order to verify preliminary results. In both cases, IHC staining showed higher signal around the calcium nodule, suggesting that both proteins are surrounding the calcified lesion. Taking into account the results from the preliminary identification and the IHC, as well the implication of both proteins in the pathology, we dare to discuss them, bearing in mind that further studies are needed, in terms of the technical identification, and trying to better understand the molecular development of the calcification in AS.

Collagen VI is a ubiquitous extracellular matrix protein which is essential in the maintenance of tissue integrity and interacts with many other extracellular matrix proteins^23^. Different collagens play an important role in CAS. Firstly, during the tissue remodeling that takes places during fibrosis development, collagen degradation by metalloproteinases is a common process^21^,^24^. This data is consistent with our results in both MALDI-IMS and IHC, in which we observed different distribution of the 4321 Da peptide which may belong to collagen VI and the full protein. Besides, localization of collagen VI around the calcified lesion could be important for the spreading of the mineralization. Several studies showed that collagens play an important role in regulating the ensuing biomineralization cascade^25^,^26^. Specifically, collagen VI is responsible for calcium and hydroxyproline accumulations on human osteoblast-like cells and may be important to osteoblastic differentiation.

Concerning NDRG-2, it is a protein with high expression in cardiac tissue but whose functions in the heart are largely unstudied. It has been shown that is involved in p53-mediated apoptosis^27^. Moreover, its upregulation due to glucose or oxygen-glucose deprivation^28^ or hypoxia^29^ produces the induction of cell apoptosis. In the context of the AV calcification, the initiation of the mineralization by the osteoblast-like cells may be stimulated by the presence of cellular degradation products following apoptosis^30^, even though, some studies demonstrated that increased apoptosis precedes calcification^22^,^31^. This seems to be in concordance with our results, where we observed more density of NDRG2 surrounding the calcified area. In fact, the administration of an apoptosis inhibitor to cultured valvular interstitial cells has been shown to results in a significant decrease in nodules calcification^22^.

To the best of our knowledge, this is the first MALDI-IMS study to investigate AS, offering the great advantage of exploring changes taking place directly in tissue. Our study paves the way for a better understanding of CAS pathophysiology and degenerative process. In recent years, great efforts have been made to understand the mechanisms implicated in degenerative CAS, nevertheless, still remains largely unclear. Indeed, the development of new biomarkers and therapies will require a clear comprehension of the pathogenic mechanisms of disease onset and progression, highlighting the necessary relationship among molecular mechanisms-biomarkers-therapies. With all this in mind, we envision that biomarkers that shall be detectable in blood and that may come from studies as the one shown here are an attractive goal in the study of CAS as two-folds: a) prediction of CAS severity could theoretically reduce the need for echocardiographic evaluation, a relatively costly diagnostic tool; and b) prediction of the particular patient disease progress could potentially allow for tailoring of follow-up visit frequency on an individual basis. Therefore, for the latter, patients identified as high risk of rapid disease progression would have frequent regular follow-up visits and those at low risk could receive less frequent follow-ups, thus allowing for optimization of health care resources. Based on these two premises and with the scope of the biomarkers, it is important to underline the on tissue putative identification and localization of collagen VI and NDRG2 fragments. These proteins, which are directly involved in the development of the lesion, could be good biomarker candidates in clinical trials for diagnosis and/or prognosis management. Even so, it would be necessary to perform extensive studies for both proteins using different kind of samples such as plasma.

In conclusion, this study allows the differentiation between different structures by MALDI-IMS and sheds light on the molecular mechanism implied in AS using this novel, sensitive and promising technique. This finding may help, not only to increase our understanding about the etiology and pathophysiology of this disease, but also in the clinical management of patients with AS. A more extensive study may allow distinguishing molecular patterns that could be clues for preventing valve calcification. Besides, the presence of these proteins in the calcified area as well as during the progression of the lesion, points towards their possible implication in the development of the calcification, although more studies are needed in order to corroborate our results.

## Methods

### Tissue sampling, sectioning and storage

Heart valves with CAS were obtained from patients who underwent AV replacement due to severe CAS. A total of 6 valves (male: 6, average age: 83, presence of hypertension: 67%) with moderate calcification were selected for the study. It should be noted that all the valves present the same differentiated regions of calcification and the same degree of injury, moderate lesion. The valves were classified from a blind macroscopically inspection by cardiovascular surgeons. Of them, 1 valve was used for MALDI-IMS optimization with 100 μm lateral resolution (data not shown). A second valve was used for MALDI-IMS experiment with a resolution of 75 μm (3 different sections at different degree of injury). Both 100 μm and 75 μm preparations showed very similar results. Finally, 4 valves were used for IHC analyses (4 different sections at different levels of the lesion).

Aortic valves were washed with PBS and immediately frozen and stored at −80 °C in order to preserve the native tissue morphology. This study was carried out in accordance with the recommendations of the Helsinki Declaration and it was approved by the ethics committee at the Hospital “Virgen de la Salud” (Toledo, Spain). Signed informed consent was obtained from all individuals prior to entry onto the study.

Aortic valve tissues were sectioned at 10 μm using a Microm HM560 cryostat (Thermo Scientific) and thaw-mounted on ITO-coated glass slides (Bruker Daltonics) for mass spectrometry (MS) analysis (Fig. 1), following previously published protocols^32^,^33^,^34^,^35^. Before sectioning, tissues were embedded in water and placed in the cryostat until water is frozen. In order to maintain tissue integrity the samples were not decalcified. Immediately after mounting the sections, the slides were desiccated for 30 min and delipidized using a standard Carnoy procedure: sequential washes of 70% ethanol for 30 s, 100% ethanol for 30 s, Carnoy’s fluid (ethanol/chloroform/acetic acid: 6:3:1) for 2 min, 100 ethanol for 30 s, water with 0.2%TFA for 30 s and a final step of 100% ethanol for 30 s^13^. After delipidization, samples were dessicated again during 20 min. Three different sections of the valve, separated by 800 μm between them, were studied. Besides, sections from the intermediate regions were also collected and processed for histological staining.

### MALDI imaging mass spectrometry

For MALDI-IMS measurements, sinapinic acid (SA) matrix [15 mg/ml in 70:30 ACN:TFA 0.1%] was sprayed using a Langartech (Ideko-IK4) custom made sprayer^36^. Standard in house spraying protocol was used, with a three-pass spraying stage followed by five-pass drying stage. Both stages were repeated ten times to achieve optimal layer deposition. Analysis of tissue sections were performed using a Bruker Daltonics Autoflex III Smartbeam MALDI-TOF/TOF mass spectrometer equipped with SmartBeam Nd:YAG/355-nm laser operating at a repetition rate of 100 Hz. Imaging data acquisitions were performed in linear geometry under optimized delayed extraction conditions, in a mass range of 1000 to 30000 Da in positive ionization mode. Instrumental parameters were set to obtain the best signal-to-noise ratio and remained constant throughout. For IMS data acquisitions, 500 shots were summed per array position with a spatial resolution of 75 μm. Software used for data acquisition was FlexControl 3.0 (Bruker Daltonics).

Data analysis was performed with FlexImaging 3.0 (Bruker Daltonics). ClinProt Tools 2.2 (Bruker Daltonics) was used to perform peak peaking and statistics. All data comparisons were performed equally, summarizing signals above a signal-to-noise ratio greater than 3.0. Routinely, all spectra are baseline corrected using Top Hat algorithm with a 10.0% minimal baseline width, and smoothed using Savitsky-Golay algorithm with a 2.0 width (m/z) and 5 cycles. All spectra were recalibrated using 1000 ppm of maximal peak shift and 30% match to calibrant peaks. For display, imaging data was loaded using FlexImaging 3.0, and a list of masses corresponding to peaks was created. For multivariate analyses, PCA and unsupervised hierarchical clustering were performed to observe the differential distribution of masses in the surface of the tissue.

### Orthogonal LC and nanoLC-MS/MS analysis

Several AV slices were pooled, exposed at 20% ACN (0.1% TFA, in water) during 1 min and centrifuged (19000 × g, 5 min). Supernatant was collected while the remaining pellet was resuspended in 70% ACN (0.1% TFA, in water) during 1 min and centrifuged. Supernatants, containing eluted peptides from the tissue, were mixed, dried and resuspended in 0.1% TFA. This sample was incorporated onto a liquid chromatograph (Agilent 1200) equipped with a High-pH RP column (Phenomenex Gemini C18 15 × 2.0 mm, 5 μm particle size, pH 10) and separated in a 60 min linear gradient (5 to 75% ACN, 500 μl/min). Aliquots were collected each 2 min and dried in a speedvac. Peptide containing aliquots were loaded onto a classical RP-nanoLC (nanoAcquity LC [Waters], with a RP BEH C18 collumn 200 × 0.75 mm, 3 μm particle size, pH 3) coupled with an Orbitrap-XL MS (Thermo). The mass spectrometer automatically switched between MS and MS/MS acquisition in DDA mode. Full MS scan survey spectra (m/z 400–2000) were acquired with mass resolution of 30000 at m/z 400. The six most intense ions above 1000 counts were sequentially subjected to collision-induced dissociation (CID) in the linear ion trap. Precursors with charge states of 2 and 3 were selected for CID. Collision energy applied to each peptide was automatically normalized as a function of m/z and charge state. To identify less abundant peptides in a sample, dynamic exclusion method was used; peptides were excluded from further analysis during 60 s. Peptides were identified by using Mascot search engine (Matrix Science) with Proteome Discoverer 1.4 software (Thermo Electron). Oxidation of methionines, acetyl (N-term), amidated (C-term), Gln → pyro-Glu (N-term Q), and Glu → pyro-Glu (N-term E) were selected as variable modifications. No enzyme was specified. 10 ppm of peptide mass tolerance and 0.5 Da fragment mass tolerance were allowed. Spectra were searched against UniprotKB/Swiss-Prot database restricted to Homo sapiens entries, and only peptides identified at a false discovery rate lower than 1% were considered.

### Histological analysis

Three consecutive sections from the AS valve analyzed by MALDI-IMS were used for the histological analysis. These sections were stained using Verhoeff-Van Giesson staining protocol for elastic fibers and oil red for lipids.

Besides the consecutive sections, we used 4 different AS valves, selecting 4 sections with increasing level of calcification of each one, for IHC analyses. In this case, sections were blocked with 10% BSA in PBS Buffer with 0.1% Tween 20 and incubated for 1 h with the primary antibodies at room temperature. Specifically, these analyses were performed with antibodies against CD68, α-actin (DAKO), NDRG2 and collagen α3 Type VI (Santa Cruz). Secondary antibody conjugated with biotin, streptavidin-peroxidase and DAB was used for all immunostainings (Mouse and Rabbit Specific HRP/DAB (ABC) Detection IHC kit, Abcam). Besides, additionally sections from 4 stenotic valves were used to study the distribution of NDRG2 and collagen α3 Type VI along the different stages of the lesion. For an impartial analysis of the DAB staining, an orthonormal transformation of the RGB images by using an ImageJ plugin based on Ruifrok and Johnston’s method for color deconvolution was performed^37^.

## Additional Information

**How to cite this article**: Mourino-Alvarez, L. *et al.* MALDI-Imaging Mass Spectrometry: a step forward in the anatomopathological characterization of stenotic aortic valve tissue. *Sci. Rep.* **6**, 27106; doi: 10.1038/srep27106 (2016).

## Supplementary Material

Supplementary Information

Supplementary Information

## Figures and Tables

**Table 1:** Peptides extracted from the tissue and identified using nLC-MS/MS.

Sequence	# PSMs	Mascot Score	Protein	Accession ID	MH^+^[Da] (LC-MS)	IMS Mass
AELQEVQITEEKPLLPGQTPEAAKEAELAAR	4	62.94	NDRG2	Q9UN36	3401.78	3398
IEEGVPQFLVLISSGKSDDEVDDPAVELKQFGVAPFTIAR	3	75.63	CO6A3	P12111	4316.26	4321

It is shown the identified sequence, the number of PSMs (Peptide Spectral Matches), the identification score (Mascot score), accession number identification (accession ID), detected mass using nLC-MS/MS, and the corresponding detected mass in MALDI-IMS.
