# Supplementary material for: MALDI-Imaging Mass Spectrometry: a step forward in the anatomopathological characterization of stenotic aortic valve tissue
Source: Sci Rep. 2016 Jun 3;6:27106. doi: 10.1038/srep27106 (PMC4891820; doi:10.1038/srep27106)
Supplement: Supplementary Information [file srep27106-s1.pdf]

TITLE: MALDI-Imaging Mass Spectrometry: a step forward in the anatomopathological characterization of stenotic aortic valve tissue.

Laura Mourino-Alvarez [a,**], Ibon Iloro [b,**], Fernando de la Cuesta[a], Mikel Azkargorta[b], Tamara Sastre-Oliva[a], Iraide Escobes[b], Luis F Lopez-Almodovar[c], Pedro L Sanchez[d,e], Harkaitz Urreta[f], Francisco Fernandez-Aviles[e], Angel Pinto[g], Luis R. Padial[h], Finn Akerström[h], Felix Elortza[b,††], Maria G. Barderas[a,††]

[a]*Department of Vascular Physiopathology, Hospital Nacional de Parapléjicos, SESCAM, Toledo, Spain.*

[b]*Proteomics Platform, CIC bioGUNE, CIBERehd, ProteoRed-ISCIII, Bizkaia Science and Technology Park, Derio, Spain*

[c]*Cardiac Surgery, Hospital Virgen de la Salud, SESCAM, Toledo, Spain.*

[d]*Department of Cardiology, Hospital Universitario de Salamanca-IBSAL, Salamanca, Spain.*

[e]*Department of Cardiology, Hospital General Universitario Gregorio Marañón, Madrid, Spain.*

[f]*IDEKO-IK4, Arriaga Industrialdea, Elgoibar, Spain*

[g]*Cardiac Surgery, Hospital General Universitario Gregorio Marañón, Madrid, Spain.*

[h]*Department of Cardiology, Hospital Virgen de la Salud, SESCAM, Toledo, Spain.*

[**]Both authors contributed equally to this work

[††]Both senior authors contributed equally to this work

Adress for correspondence

M.G. Barderas, Laboratorio de Fisiopatologia Vascular, Edificio de Terapia 2ª planta, Hospital Nacional de Paraplejicos, SESCAM, 45071 Toledo, España. e-mail: megonzalezb@sescam.jccm.es FAX: 925247745.

Felix Elortza, CIC bioGUNE, Bizkaia Science and Technology Park, Building 800, 48160, Derio, Spain; Email: felortza@cicbiogune.es. FAX: +34 946572502

Fig. SM1 Histological staining of the 3 different sections analyzed using MALDI-IMS, from lesser to greatest degree of the lesion (A, B and C). From left to right: elastic fibers staining, lipids staining, myofibroblasts (α-actin) and macrophages (CD68) histoimmunochemistry. A characteristic area of each case is enlarged, showing calcification (triangle), elastic fibers and collagen (continuous and discontinuous black arrows, respectively) myofibroblasts (blue arrows) and macrophages (green arrows).

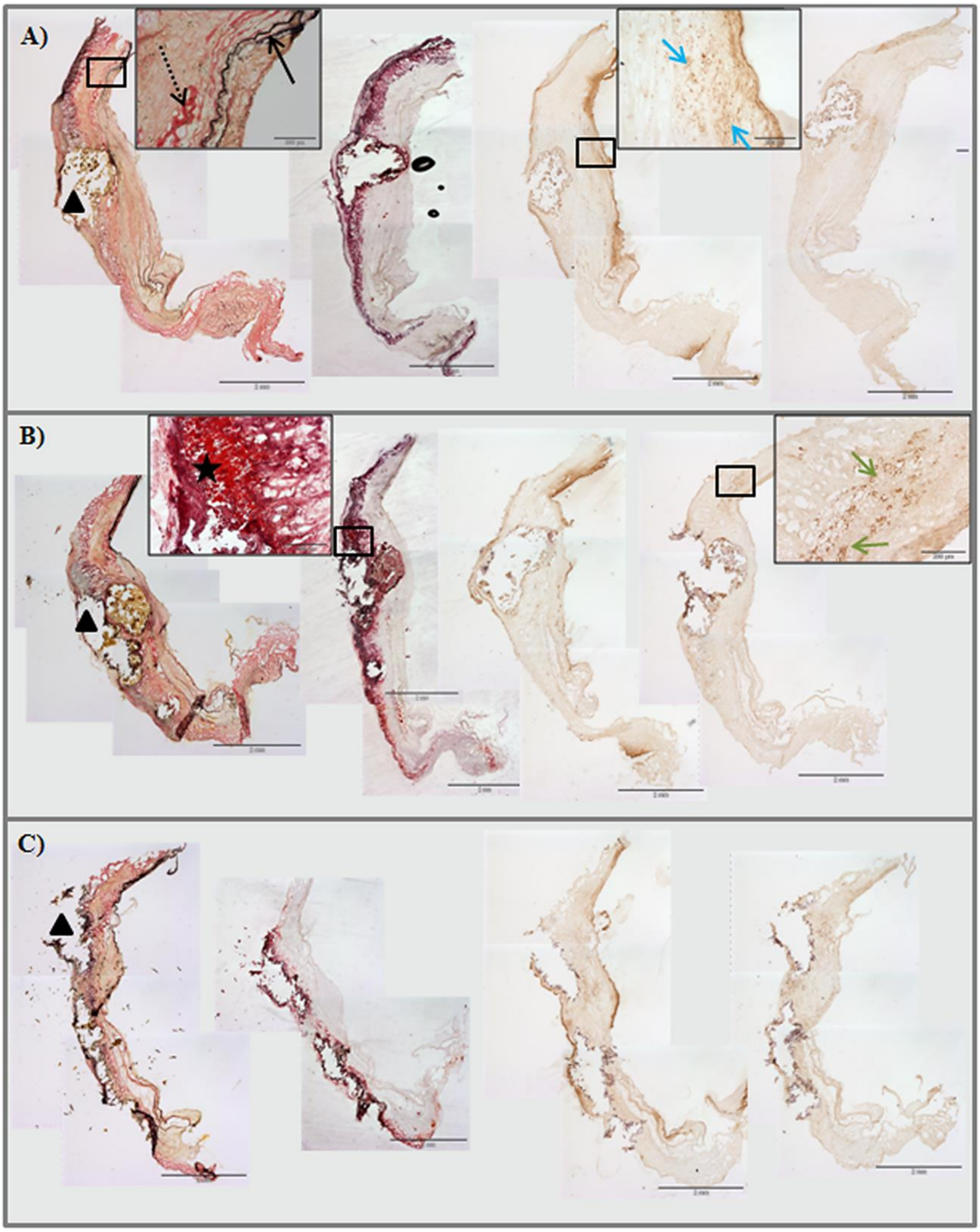
A)
B)
C)
