# Supplementary figures and images for: MALDI-Imaging Mass Spectrometry: a step forward in the anatomopathological characterization of stenotic aortic valve tissue
Source: Sci Rep. 2016 Jun 3;6:27106. doi: 10.1038/srep27106 (PMC4891820; doi:10.1038/srep27106)

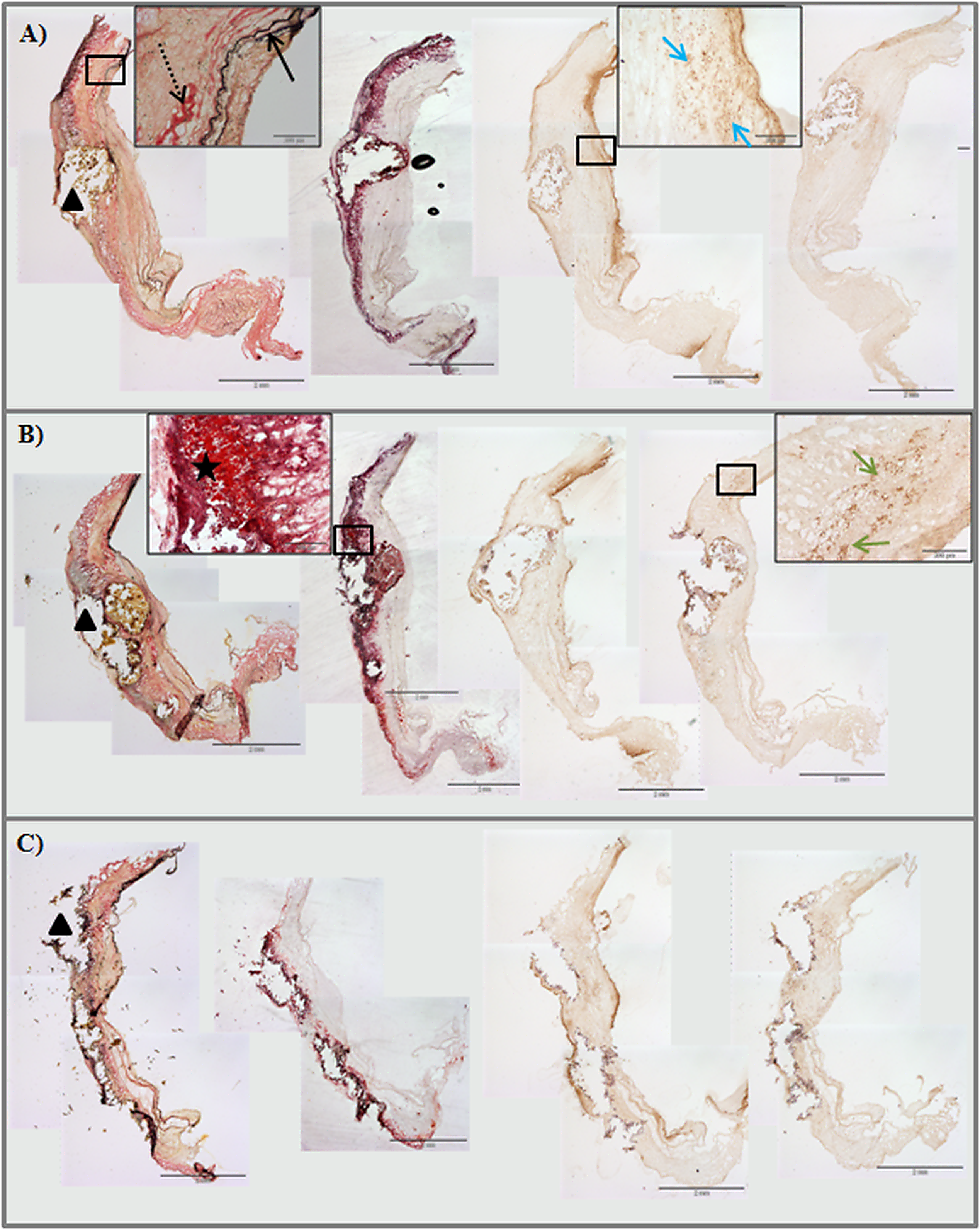

Supplement: Supplementary Information [file srep27106-s2.tiff]
